# The Interaction of Vitamin D and Corticosteroids: A Mortality Analysis of 26,508 Veterans Who Tested Positive for SARS-CoV-2

**DOI:** 10.3390/ijerph19010447

**Published:** 2021-12-31

**Authors:** Jimmy T. Efird, Ethan J. Anderson, Charulata Jindal, Thomas S. Redding, Andrew D. Thompson, Ashlyn M. Press, Julie Upchurch, Christina D. Williams, Yuk Ming Choi, Ayako Suzuki

**Affiliations:** 1 Cooperative Studies Program Epidemiology Center, Durham VA Health Care System, Durham, NC 27705, USA; thomas.redding28@va.gov (T.S.R.); andrew.thompson3@va.gov (A.D.T.); ashlyn.press@va.gov (A.M.P.); julie.upchurch@va.gov (J.U.); christina.williams4@va.gov (C.D.W.); ayako.suzuki@duke.edu (A.S.); 2 College of Pharmacy, University of Iowa, Iowa City, IA 52242, USA; ethan-anderson@uiowa.edu; 3 Harvard Medical School, Harvard University, Boston, MA 02115, USA; charujindal@gmail.com; 4 Department of Medicine, Duke University, Durham, NC 27710, USA; 5 Duke Cancer Institute, Duke University, Durham, NC 27710, USA; 6 Signify Health, Dallas, TX 75244, USA; ychoi@signifyhealth.com; 7 Division of Gastroenterology, Duke University, Durham, NC 27710, USA; 8 The Division of Gastroenterology, Durham VA Medical Center, Durham, NC 27705, USA

**Keywords:** anti-inflammatory, corticosteroids, COVID-19, cytokine storm, SARS-CoV-2, vitamin D, veterans

## Abstract

This data-based cohort consisted of 26,508 (7%) United States veterans out of the 399,290 who tested positive for SARS-CoV-2 from 1 March to 10 September 2020. We aimed to assess the interaction of post-index vitamin D (Vit D) and corticosteroid (CRT) use on 30-day mortality among hospitalized and non-hospitalized patients with coronavirus disease 2019 (COVID-19). Combination Vit D and CRT drug use was assessed according to four multinomial pairs (−|+, −|−, +|+, +|−). Respective categorical effects were computed on a log-binomial scale as adjusted relative risk (aRR). Approximately 6% of veterans who tested positive for SARS-CoV-2 died within 30 days of their index date. Among hospitalized patients, a significantly decreased aRR was observed for the use of Vit D in the absence of CRTs relative to patients who received CRTs but not Vit D (aRR = 0.30; multiplicity corrected, *p* = 0.0004). Among patients receiving systemically administered CRTs (e.g., dexamethasone), the use of Vit D was associated with fewer deaths in hospitalized patients (aRR = 0.51) compared with non-hospitalized patients (aRR = 2.5) (*P*-for-Interaction = 0.0071). Evaluating the effect of modification of these compounds in the context of hospitalization may aid in the management of COVID-19 and provide a better understanding of the pathophysiological mechanisms underlying this and future infectious disease outbreaks.

## 1. Introduction

Vitamin D (Vit D) is an oxysterol hormone with important immunomodulatory and antiviral properties [1,2,3,4,5,6,7,8]. Even though this vitamin is often recommended as a nutritional supplement in treatment guidelines and prescribed for the management of coronavirus disease 2019 (COVID-19) [9,10,11,12], several studies have yielded divergent or inconclusive results [13,14,15,16,17,18,19,20,21,22,23,24,25,26,27,28,29,30,31,32,33,34,35,36,37,38,39,40,41,42,43,44,45,46,47,48,49,50,51,52,53,54].

The complex interaction of Vit D and corticosteroids (CRTs) in vivo may partially explain the conflicting outcomes observed in the COVID-19 literature, and previous studies on Vit D have been insufficiently powered to detect this interaction [55,56,57,58,59]. Glucocorticoids can inactivate Vit D by upregulating Vit D receptor (VDR)-mediated 24-hydroxylase transcription through the cooperative relationship of CCAAT-enhancer-binding proteins (C/EBPβ) and glucocorticoid receptors (GRs) [60]. This mechanism is likely the reason why CRT use in a nationally representative cohort study was associated with a two-fold reduction in endogenous Vit D levels [61]. Indeed, suboptimal concentrations of serum 25-hydroxyvitamin D3 (25(OH)D_3_) in the order of −0.5 ng/mL have been linked with the use of glucocorticoids such as dexamethasone, methylprednisolone, and prednisone [62,63].

However, these Vit D-lowering effects of CRTs may be countered by the fact that clinical doses of dexamethasone enhance the effects of 25(OH)D_3_, thus inducing VDR expression in immune cells [64,65,66]. Administration of Vit D purportedly reverses the induction of interleukin 10 (IL-10)-secreting regulatory T cells in glucocorticoid-resistant patients, a mechanism particularly beneficial in the context of COVID-19 [67]. Vit D has also been shown to have a synergistic anti-inflammatory effect with CRTs by facilitating glucocorticoid induction of mitogen-activated protein kinase (MAPK) phosphatase 1 (MKP-1) and IL-10 in peripheral blood mononuclear cells (PBMCs) [68]. Granulocyte-macrophage colony-stimulating factor (GM-CSF) found in culture supernatants from clusters of differentiation 14-negative (CD14^-^) cells and mediator complex subunit 14 (MED14) have been recognized as significant factors in this process, effectively reducing the dose of glucocorticoids needed to mitigate inflammatory effects [69]. Concurrently with CRTs, Vit D is thought to prompt a tolerogenic dendritic cell phenotype with immunomodulatory action [70,71].

The results of a meta-analysis suggest that Vit D supplementation (varying doses and frequencies across studies) improves COVID-19 clinical response, but only in patients receiving the drug after diagnosis of COVID-19 [21]. Systemic CRT use increased by 19% among hospitalized adults during the COVID-19 pandemic based on evidence that these compounds reduced 28-day mortality in hospitalized patients requiring supplemental oxygen or mechanical ventilation [72,73]. However, among those not receiving respiratory support, the administration of CRTs was consistent with possible deleterious effects. That is, if CRTs are delivered when control of viral replication is critical, blunting the inflammatory response may be more harmful than helpful [73].

**Figure 1:**
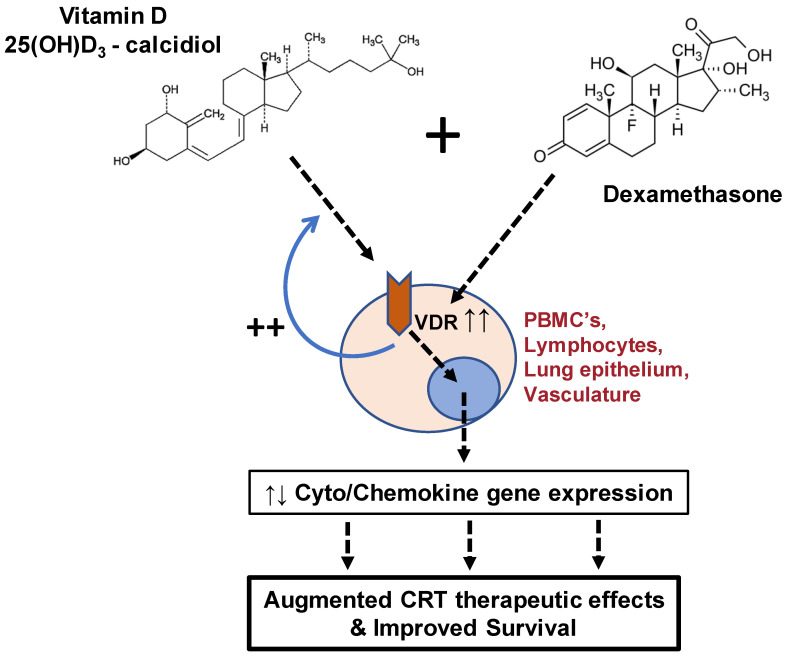
Interaction and outcomes of vitamin D and corticosteroid administration in COVID-19 patients. A cursory overview of various mechanisms by which corticosteroids (i.e., dexamethasone) and vitamin D interact synergistically in patients with COVID-19 is shown in the schematic above, along with the outcomes of this interaction as reported in the present study. CRT = corticosteroid; PBMCs = human peripheral blood mononuclear cells; VDR = vitamin D receptor; 25(OH)D_3_ = 25-hydroxyvitamin D3.

In this observational analysis, we hypothesized that Vit D and CRTs interact through various putative mechanisms to affect overall 30-day mortality among patients testing positive for SARS-CoV-2 (Figure 1). Furthermore, this effect is postulated to be dependent on hospitalization status as a surrogate marker of disease severity.

## 2. Materials and Methods

### 2.1. Study Design and Data Source

Data for this study were obtained from the United States (US) Department of Veterans Affairs (VA) COVID-19 Shared Data Resource (CSDR) and related data in the national Corporate Data Warehouse (CDW), with details described in our previous publication [74]. The VA Health Care System consists of ~171 medical centers and 1112 outpatient sites of care. Approval for the study protocol was provided by the Durham VA Health Care System Institutional Review Board.

### 2.2. Cohort Definition

The cohort consisted of veterans who tested positive for SARS-CoV-2 from 1 March to 10 September 2020. During the study period, COVID-19 testing was restricted to participants who were symptomatic for COVID-19 or were required to have a SARS-CoV-2 test for hospitalization. Hospitalized and non-hospitalized patients, males and females, and all race/ethnicity categories were included in this analysis to increase the generalizability of the study findings. We report the first SARS-CoV-2-positive collection date as the index date and only considered this first event.

### 2.3. Case Definition

Cases were identified using the VA CSDR [75]. We defined SARS-CoV-2-positive veterans using all available SARS-CoV-2 nucleic acid amplification (NAAT) and antigen tests, excluding antibody tests. Some patients in the VA Health Care System were tested or diagnosed outside of the VA, with special informatics tools used to aid the identification of COVID-19 cases [76]. However, we did not include these patients in the analyses focusing on veterans who were tested at any of the VA facilities.

### 2.4. Study Variables

The primary outcome variable was 30-day overall mortality after the first positive SARS-CoV-2 test. Hospitalizations were defined as occurring in the post-index period and typically entailed patients who experienced severe disease presentation, requiring close monitoring and intensive care [77].

Electronic outpatient and inpatient prescription and dispense records were reviewed to determine the systemic use of Vit D and CRTs. By consensus of our clinical team, the post-index use of these compounds was defined as drugs administered at least 50% of the time (≥7 days or half of the survival time) within 2 weeks post-SARS-CoV-2 testing. Length of hospital stay corresponded to only the first hospitalization happening within 30 days after the SARS-CoV-2 test date. Information and definitions regarding the covariates used in the analyses were published previously [74].

Non-VA medications, including prescriptions from non-VA providers and over-the-counter medications, were recorded in the electronic health records through medication reconciliation performed at clinical encounters. Among non-hospitalized patients, Vit D supplements were either from VA prescriptions, non-VA prescriptions, or over-the-counter supplements. We counted all available Vit D supplements, including calcitriol. Inhaled CRTs such as beclomethasone, budesonide, ciclesonide, flunisolide, fluticasone, or mometasone were not considered in the current analysis.

### 2.5. Statistical Analysis

Study characteristics are expressed as frequency and percentage for categorical variables and median and interquartile range (IQR) for continuous variables. A weighted average of stratum-specific estimates on the log-binomial scale was used to approximate the adjusted relative risk (aRR) for 30-day mortality [78]. Final models were adjusted for age categories, race, sex, region, month of diagnosis, body mass index (BMI) categories, current smoking, alcohol use disorder, and Charlson Comorbidity Index score categories. An unrestricted additive test for interaction was used to test for heterogeneity of relative effect estimates (Δ_aRR_%) on the logarithmic scale [79,80]. Vertical and horizontal interactions were similarly assessed, appropriately adjusting confidence intervals to account for the two estimates of Δ_aRR_% [81].

To assess the multinomial synergistic drug effects for Vit D and CRT, we created four categories for each pair to respectively denote single and combination drug use (−|+, −|−, +|+, +|−). The referent group (−|+) was used in our key analyses to illustrate the maximum difference in effect sizes and linearity of these effects among the hospitalized versus non-hospitalized patients. However, selected comparisons also were conducted with (−|−) as the referent group. Multiplicity correction (MC) was performed using the Hochberg–Bonferroni procedure, accounting for a common referent group [82].

The expectation-maximization algorithm was used to find maximum a posteriori estimates of model parameters, dependent on unobserved latent variables [83]. Linearization and normalization of data elements were performed when appropriate. Study results were rounded using the Goldilocks (Efron–Whittemore) method [80]. *p*-values < 0.05 were considered statistically significant. All the analyses were performed using SAS version 9.4 (SAS Institute, Cary, NC, USA).

## 3. Results

A total of 26,508 veterans tested positive for SARS-CoV-2, with a 30-day mortality rate of 6% (Table 1). Approximately 93% of deaths occurred among those >60 years of age. Most participants were male (89%), non-Latinx (83%), overweight (82%), White (59%), and ≤ 60 years of age (52%). Few patients were hospitalized for more than 2 weeks (7%) and less than 5% received mechanical ventilation. Commonly reported comorbidities were hypertension (55%), hyperlipidemia (52%), and mental illness (49%). Slightly more Black veterans than White veterans received post-index Vit D (5% versus 3%) and, similarly, CRTs (23% versus 20%). Only 1% of the cohort received a combination of Vit D and CRTs. Approximately 76% of the sample did not use either drug.

Compared with the use of post-index CRT use alone (−|+), the combination therapy of Vit D and CRTs (+|+) as well as the use of post-index Vit D alone (+|−) were logarithmically associated with 98% (*P*_mc_ = 0.031) and 223% (*P*_mc_ = 0.0004) reduced 30-day mortality adjusted risk among those hospitalized, respectively (Table 2). For this group of hospitalized patients, an aRR of 1.5 (95% CI = 1.3–1.7; *P*_mc_ < 0.0001) was observed for CRT use alone (−|+) compared with the use of neither compound (−|−) (not shown in tables). Analogously, the aRR for Vit D use alone (+|−) versus neither compound (−|−) was 0.47 (95% CI = 0.25–0.90; *P*_mc_ = 0.022).

Among non-hospitalized patients, Vit D use alone showed a tendency toward a decreased risk (aRR = 0.48, 95% CI = 0.22–1.1, *P*_mc_ = 0.078) (Table 2). A borderline significant risk increase was observed for the combined use of Vit D and CRTs (+|+) relative to the use of CRTs without Vit D (−|+) (aRR = 2.5, 95% CI = 0.90–7.1; *P*_mc_ = 0.078). Comparable risk effects were observed for the subset of Black and White hospitalized patients (Table 3 and Table 4).

A significant mortality interaction was observed for the effect of Vit D use in hospitalized versus non-hospitalized patients who received post-index CRTs (*P*-for-interaction (*P_Int_*) = 0.0071); post-index Vit D use showed a protective effect among hospitalized post-index CRT users but a borderline detrimental effect among non-hospitalized post-index CRT users (Table 5). This interaction remained statistically significant after multiplicity correction. Among non-users of post-index CRTs, post-index Vit D use showed a protective effect, regardless of hospitalization. The Δ_aRR_% (Vit D versus no Vit D) for non-users of post-index CRTs was −8, corresponding to a borderline significant vertical interaction compared with users of post-index corticosteroids (*P*_ΔV_ = 0.057 before MC). In contrast, among those not receiving post-index Vit D, a 50% increased aRR (30-day mortality) was observed for hospitalized users of post-index corticosteroids (versus non-users) (*p* < 0.0001), and this represented a Δ_aRR_% of +43 compared with non-hospitalized patients (*p* = 0.018 before MC). However, there was no evidence of either a vertical (*P*_ΔV_ = 0.81) or horizontal (*P*_ΔH_ = 0.53) interaction for the CRT comparisons.

## 4. Discussion

Considerable discordance exists regarding the effectiveness of Vit D in the treatment of COVID-19, with some reports suggesting a beneficial effect [15,16,17,28,29,31,41,45,53,84,85,86,87,88,89,90,91,92,93,94,95,96,97,98,99,100,101,102,103,104,105,106] and others no change [26,27,30,32,33,34,35,36,37,44,107,108,109]. The reasons for these disparate findings are likely complex and multifactorial, with varying doses, duration, and timing of Vit D administration along with patient populations and sample sizes all playing roles [110]. Differences in the criteria for defining the severity of COVID-19 may also explain, in part, the ambiguity of study results [111]. Most of the studies on this topic have been observational in design and did not account for heterogeneous comorbid conditions or the effect of CRT use. Moreover, the few randomized clinical trials published to date have been small and insufficiently powered to assure the balance of CRT use between study arms [35,36,84,86,96].

Our findings suggest that post-index use of Vit D and CRT (+|+) has a beneficial effect on survival among hospitalized patients with PCR-confirmed COVID-19. Considering hospitalization to be a surrogate marker for disease severity and, in many cases, the need for respiratory support, the current analysis is consistent with the RECOVERY Collaborative Group findings [73]. In the latter study, dexamethasone was associated with a harmful effect when administered to patients not receiving respiratory support, and vice versa. Accordingly, if Vit D enhances the effect of CRT, as suggested by several reports, it follows that Vit D + CRT would similarly have an increased benefit during hospitalization, when the control of inflammation versus viral replication is paramount [58,59,60,64,65,66,67,70,71]. The highest mortality risk in our study was noted for hospitalized patients receiving CRTs in the absence of Vit D. In contrast, the combination of Vit D and CRT significantly reduced this risk by 98% on the logarithmic scale.

As previously mentioned, CRT use in a nationally representative cohort study was associated with a two-fold reduction in endogenous Vit D levels [61]. Assuming that Vit D has a positive effect on survival, those in the CRT-only treatment group (−|+), as expected and consistent with our findings, were at a survival disadvantage. Nonetheless, we cannot rule out a potentially detrimental effect of CRTs by suppressing the immune response to clear the virus.

In response to viral infection, CRTs inhibit inflammation by impeding endovascular L-selectin synthesis and through the release of granulocytes from bone marrow [112,113,114,115,116,117,118,119]. Systemic glucocorticoids have been demonstrated to improve survival when administered to COVID-19 patients who are moderately or critically ill, but their effectiveness is dependent upon timing, dose, and patient-level characteristics [73,120]. Perhaps equally important in identifying the best candidate for CRT therapy, as put forward by our findings, is the interaction of Vit D with CRT, especially in the context of hospitalization. Future confirmation of this hypothesis in the form of sufficiently powered randomized controlled trials is needed, although this may not be practical given the increasing rates of immunity among the population conveyed by vaccination. However, Vit D and CRT may yet be beneficial for the treatment of COVID-19 patients infected with emerging variants as the SARS-CoV-2 virus evolves over time [121].

Given the recent results of the randomized placebo-controlled trial of a one-time bolus of high-dose Vit D, which showed no improvement in hospital length of stay in COVID-19 patients [36], it is noteworthy that Vit D use by patients in our study was generally low-dose daily administration from days to weeks post-COVID-19 diagnosis. There is ample evidence that chronic, daily Vit D dosing is superior to bolus high-dose administration for many reasons, several of which have been summarized in recent publications [122,123,124]. For example, a single high dose of Vit D, versus daily administration, has been reported to activate the 24-hydroxylase enzyme (CYP24A1), leading to increased production of the inactive form of Vit D (i.e., 24,25(OH)_2_D_3_) [125]. High-dose Vit D may also suppress parathyroid hormone (PTH) through its effect on parathyroid cells [126]. PTH is an immunomodulator postulated to stimulate the phytohemagglutinin (PHA)-induced proliferation of T cells, important for cell-mediated immunity in the fight against infection [127,128,129]. In addition to T cells, receptors for PTH are found on neutrophils and B cells of the immune system [130]. Nonetheless, the treatment response dynamics of Vit D are complex and nonlinear, especially with respect to PTH [131].

Patients in the aforementioned clinical trial were excluded if they required invasive mechanical ventilation; most did not need noninvasive ventilation, suggesting less severe illness. Notably, CRT use has the greatest benefit among severely ill COVID-19 patients, with those being moderately ill manifesting little or no improvement [132]. Additionally, CRT use was not balanced between the study arms of this trial, thus hindering the interpretation of the reported results.

Our study was sufficiently powered to detect drug–drug interactions. Indeed, an important strength of this study is that the Veterans Health Administration (VHA) represents the largest integrated health care system in the US, with reasonably complete and up-to-date electronic database information for demographics and medication prescription details [133,134]. Additionally, given the longitudinal frame of the database, mortality risk on a log-binomial scale was directly estimated, thus minimizing inflated effect sizes associated with odds ratios.

A few limitations should be considered when evaluating our findings. First and foremost, this was not a randomized clinical trial. The potential for undiagnosed (untested) seropositivity owing to mild or asymptomatic cases may have biased the study results if a differential effect existed by hospitalization status. However, this bias is likely nominal given that our models were adjusted for key outcome-related variables, including the Charlson Comorbidity Index. Nonetheless, the potential remains for residual confounding, misclassification bias, collider effects, and hidden interactions among the analyzed variables as well as variables not included in our multivariable models (e.g., social determinants of health, quality of life indicators, and inability to self-isolate or manage care at home) [135].

Incomplete information on inactive users and veterans tested and treated outside the VHA health care system are other important sources of study bias unaccounted for in our analysis [136]. We also cannot rule out ascertainment bias, especially during the initial phase of the pandemic, with hospitalized patients being more likely to have high-risk conditions such as hypertension, diabetes, or renal impairment [136]. However, our analyses were adjusted for time and comorbidities. Differences in testing rates for COVID-19 have been reported in the literature, with non-White individuals generally being disproportionately represented among those infected, hospitalized, and deceased, and they similarly tend to be younger and have increased comorbid conditions [137]. In some reports, specific occupations such as health care workers were also tested more often than the general population [138]. Although the VA is generally recognized as an equal access system, study bias among ill versus healthy participants must be acknowledged if this resulted in higher or lower utilization and hospitalization rates among certain patients. Moreover, our study relied solely on SARS-CoV-2 testing to determine COVID-19 status rather than clinical adjudication.

Information on non-VA medications is based on self-reporting and may potentially have been underreported. We were unable to confirm adherence and compliance to medications among non-hospitalized patients, and this possibly introduced a variance with respect to those who were hospitalized. Owing to differences in documentation and drug nomenclature across VA clinics, determining the exact dosing and duration of Vit D and CRTs was beyond the scope of the current study. For analogous reasons, the effect of polypharmacy and the interaction with nutrients such as zinc, selenium, and vitamin C on mortality was not evaluated in our analyses. We also did not consider the combination of Vit D with medications such as orlistat, statins, and thiazide diuretics, which are known to affect Vit D levels [139]. Although important in the natural history of COVID-19, changing mitigation strategies and restrictions likewise were not studied [140,141]. Furthermore, the potential for differential health-seeking behaviors should be noted as a possible limitation [142].

Protopathic bias is another factor to consider when evaluating our findings. Levels of Vit D have been reported to precipitously drop during the initial, acute inflammatory phase of critical illness [109]. Conceivably, severe COVID-19 may manifest as lowered Vit D levels, in turn necessitating the need for this vitamin among hospitalized patients. However, this potential reverse causality effect would not explain the interaction with CRTs or the multinomial results observed in our study. Indeed, the opposite appears to be the case in that the mortality risk among hospitalized patients receiving both Vit D and CRTs was statistically lower than that for patients using CRTs in the absence of Vit D. While hospitalization status was considered a surrogate marker of disease severity in our analysis, we recognize that different patients might have presented to the hospital with varying states of acute illness [109]. We also acknowledge that the response to Vit D in patients with already high levels at the time of hospital admission may be more beneficial than efforts aimed at increasing levels within a brief window with treatment [105]. In future analyses, we will aim to delineate individual dose levels by model covariates and outcome variables.

Our sample, while reflective of the VHA population, consisted mainly of older males, with only 11% being female [133,134,140,141]. Patients treated within the VHA tend to be at higher risk with more medically complex conditions than the US population [143]. Veterans, especially those with obesity, diabetes, cardiac diseases, and a history of military exposures, may be more susceptible to COVID-19-induced inflammatory conditions and poor recovery from the disease owing to decreased reserve and pre-existing endothelial dysfunction (these conditions, which have been linked to Vit D deficiency, suggest a potential benefit of Vit D to augment host response to COVID-19) [144,145,146]. In Hawaii, for example, where sunlight is abundant, veterans newly admitted to a nursing home were observed to have a very high prevalence of Vit D deficiency [147], which was presumably higher than that for non-veterans. Hence, we cannot guarantee that our results are generalizable to other health care systems, even after carefully adjusting for key outcome-related confounders. Lastly, our study did not consider polymorphisms of Vit D metabolism and Vit D binding protein when analyzing the interaction of Vit D and CRT use [148,149].

Independent of COVID-19, lower levels of 25-hydroxyvitamin D (25D) on day 1 of intensive care unit (ICU) admission have been associated with decreased production of cathelicidin antimicrobial protein-18 (hCAP-18) and greater mortality risk at three months [132]. Patients in the ICU are, in general, at risk of having lower levels of 25-hydroxyvitamin D (25D) and suboptimal cellular oxygenation for various reasons (e.g., fluid resuscitation, renal failure, cardiac/respiratory failure, and gastrointestinal bleeding) [150]. When administered in this setting, Vit D replacement therapy may, in theory, reduce mortality by increasing hemoglobulin levels, decreasing hepcidin concentrations, and facilitating oxygenation at the cellular level [105]. Thus, aspects of Vit D use noted in the current paper may also apply overall to hospitalized patients with severe illness, warranting a study of Vit D in this broader population while considering a possible interaction with CRT use in the study design.

The use of Vit D in excess amounts (>50,000 IU per day) is toxic and may lead to hypercalcemia and hyperphosphatemia [139,150]. Accordingly, patients are advised to avoid high doses of Vit D for the prevention or treatment of COVID-19 unless prescribed by a licensed health care provider.

## 5. Conclusions

In accordance with our hypothesis and the results from this large observational study, there appears to be an interaction between post-index use of Vit D and CRT with respect to 30-day mortality among hospitalized versus non-hospitalized patients testing positive for SARS-CoV-2. Future independent analyses are needed to validate this effect and the importance to both clinical practice and our understanding of COVID-19 pathophysiology.

## Figures and Tables

**Table 1:** Selected characteristics of SARS-CoV-2-positive veterans (*N* = 26,508).

Characteristic	Post-Index Medication Usage * (Vitamin D|Corticosteroids) n (%), Median (IQR)				30-Day Mortality n (%), Median (IQR)
	(−|+)	(−|−)	(+|+)	(+|−)	
N (% of total sample size)	5355 (20)	20,134 (76)	283 (1)	736 (3)	1612 (6)
Age (y)	64 (21)	58 (27)	66 (16)	63 (20)	76 (16)
≤30	154 (3)	1425 (7)	5 (2)	14 (2)	0 (0)
31–40	455 (9)	3058 (15)	12 (4)	59 (8)	3 (<1)
41–50	598 (11)	2725 (14)	26 (9)	84 (11)	25 (2)
51–60	976 (18)	3864 (19)	49 (17)	165 (22)	80 (5)
61–70	1362 (25)	4101 (20)	88 (31)	179 (24)	328 (20)
71–80	1301 (24)	3410 (17)	76 (27)	177 (24)	569 (35)
81–90	401 (7)	1151 (6)	19 (7)	37 (5)	401 (25)
>90	108 (2)	400 (2)	8 (3)	21 (3)	206 (13)
Male ^^^	4831 (90)	17,970 (89)	240 (85)	618 (84)	1575 (98)
Race					
White	3047 (57)	12,070 (60)	147 (52)	392 (53)	1009 (63)
Black	2101 (39)	7257 (36)	129 (46)	328 (45)	547 (34)
Asian	59 (1)	226 (1)	1 (<1)	3 (<1)	15 (1)
AIAN	50 (1)	166 (1)	0 (0)	2 (<1)	16 (1)
NHOPI	51 (1)	207 (1)	2 (1)	7 (1)	13 (1)
Multiracial	47 (1)	208 (1)	4 (1)	4 (1)	12 (1)
Latinx ^^^	790 (15)	3536 (18)	25 (9)	102 (14)	154 (10)
BMI (kg/m^2^)	31 [8.3]	30 [7.7]	30 [8.5]	30 [8.6]	27 [9]
Underweight (<18.5)	100 (2)	243 (1)	7 (2)	10 (1)	73 (5)
Normal (18.5–24.9)	883 (16)	3403 (17)	45 (16)	127 (17)	489 (30)
Overweight (25–29.9)	1655 (31)	6678 (33)	83 (29)	249 (34)	490 (30)
Class-I Obese (30–34.9)	1462 (27)	5748 (29)	90 (32)	178 (24)	304 (19)
Class-II Obese (35–39.9)	777 (15)	2614 (13)	34 (12)	104 (14)	159 (10)
Class-III Obese (40–44.9)	323 (6)	984 (5)	18 (6)	35 (5)	55 (3)
Super Obese (≥45)	155 (3)	464 (2)	6 (2)	33 (4)	42 (3)
Alcohol Use Disorder ^^^	671 (13)	2683 (13)	37 (13)	118 (16)	163 (10)
Smoker ^§^					
Never	2413 (45)	10,167 (51)	137 (48)	381 (52)	644 (40)
Former	2406 (45)	7623 (38)	118 (42)	297 (40)	858 (53)
Current	536 (10)	2344 (12)	28 (10)	58 (8)	110 (7)
Hospitalization ^^^	3149 (59)	4340 (22)	164 (58)	192 (26)	1113 (69)
Length of Stay (days)	8 [11]	6 [10]	6 [12]	5 [11]	9 [9]
≤7 ^~^	3751 (70)	18,398 (91)	209 (74)	669 (91)	946 (59)
>7–14	790 (15)	854 (4)	29 (10)	28 (4)	388 (24)
>14	814 (15)	882 (4)	45 (16)	39 (5)	278 (17)
Mechanical Ventilation ^^^	673 (13)	465 (2)	30 (11)	14 (2)	598 (37)
Location (USA)					
Pacific-West/Mountain	971 (18)	3996 (20)	42 (15)	97 (13)	231 (14)
Mid-West/Continental	1053 (20)	4175 (21)	42 (15)	93 (13)	251 (16)
Southeast	2598 (49)	8111 (40)	180 (64)	442 (60)	592 (37)
Northeast	733 (14)	3852 (19)	19 (7)	104 (14)	538 (33)
Time (Index, 3/1–9/10)					
March	362 (7)	1668 (8)	17 (6)	54 (7)	253 (16)
April	512 (10)	2916 (14)	26 (9)	97 (13)	445 (28)
May	297 (6)	1730 (9)	12 (4)	61 (8)	190 (12)
June	953 (18)	3592 (18)	42 (15)	122 (17)	205 (13)
July	2155 (40)	7136 (35)	114 (40)	277 (38)	315 (20)
August	998 (19)	2950 (15)	68 (24)	121 (16)	192 (12)
September	78 (1)	142 (1)	4 (1)	4 (1)	12 (<1)
CCI					
0	2198 (41)	11,712 (58)	93 (33)	329 (45)	407 (25)
1–2	1978 (37)	6032 (30)	115 (41)	291 (40)	572 (35)
3–4	766 (14)	1640 (8)	48 (17)	77 (10)	391 (24)
5^+^	413 (8)	750 (4)	27 (10)	39 (5)	242 (15)
Comorbidity ^^^					
Asthma	591 (11)	1039 (5)	29 (10)	56 (8)	74 (5)
Atherosclerosis	1830 (34)	4551 (23)	112 (40)	207 (28)	872 (54)
Cancer	885 (17)	2095 (10)	80 (28)	137 (19)	391 (24)
Chronic Kidney Disease	1173 (22)	2664 (13)	77 (27)	121 (16)	612 (38)
Chronic Liver Disease	145 (3)	408 (2)	13 (5)	22 (3)	68 (4)
CHF	900 (17)	1930 (10)	52 (18)	89 (12)	452 (28)
COPD	1307 (24)	2188 (11)	79 (28)	108 (15)	472 (29)
Diabetes (Type II)	2108 (39)	6116 (30)	122 (43)	307 (42)	813 (50)
Hyperlipidemia	3227 (60)	10,032 (50)	183 (65)	466 (63)	1047 (65)
Hypertension	3523 (66)	10,461 (52)	209 (74)	501 (68)	1242 (77)
Mental Illness	2716 (51)	9662 (48)	148 (52)	409 (56)	709 (44)
Sleep Disorder	1828 (34)	5111 (25)	99 (35)	214 (29)	416 (26)
Substance Abuse	1194 (22)	4222 (21)	62 (22)	173 (24)	307 (19)

^^^ Referent is the complement group. * Systemic administration by mouth or intramuscular injection. ^§^ Cigarettes. ^~^ Includes non-hospitalized participants with zero length of stay. AIAN = American Indian and Alaska Native; BMI = body mass index; CCI = Charlson Comorbidity Index; CHF = congestive heart failure; COPD = chronic obstructive pulmonary disease; IQR = interquartile range; kg = kilograms; m = meters; NHOPI = Native Hawaiian or Pacific Islander; USA = United States of America; y = years.

**Table 2:** Adjusted risk for non-survivors and survivors among SARS-CoV-2-positive veterans by indicated post-index medication use and hospitalization status.

Post-Index		Non Survivors ^^^	Survivors ^^^	Multiplicity Corrected ^‡^	
Medication *		n (%)	n (%)	aRR (95% CI) ^†^	*p*-Value
Hospitalized (*N* = 7845)					
Vitamin D	Corticosteroids				
−	+	534 (48)	2615 (39)	1.0 Referent	---
−	−	553 (50)	3787 (56)	0.66 (0.58–0.74)	<0.0001
+	+	15 (1)	149 (2)	0.51 (0.27–0.94)	0.031
+	–	11 (1)	181 (3)	0.30 (0.16–0.58)	0.0004
Non-hospitalized (*N* = 18,663)					
Vitamin D	Corticosteroids				
−	+	69 (14)	2137 (12)	1.0 Referent	---
−	−	413 (83)	15,381 (85)	0.94 (0.71–1.2)	0.66
+	+	6 (1)	113 (1)	2.5 (0.90–7.1)	0.078
+	–	11 (2)	533 (3)	0.48 (0.22–1.1)	0.078

^^^ Non-referent group of the indicated comparison factor. * Systemic administration by mouth or intramuscular injection. ^†^ Adjusted for Age (≤60, 61–70, 71–80, >80), Alcohol Use Disorder (Yes, No), BMI (<18.5, 18.5–24.9, 25–29.9, ≥30), Charlson Comorbidity Index (0, 1–2, 3–4, 5^+^), Current Smoker (Yes, No), Location (Pacific-Mountain, Mid-West/Continental, East Coast), Race (White, Black, Other Race), Sex (Male, Female), and Time (March, April–September). ^‡^ Subset analyses corrected for multiplicity using the Hochberg step-up procedure for multinomial comparisons. aRR = Adjusted relative risk; BMI = body mass index (kg/m^2^); CI = confidence interval.

**Table 3:** Adjusted risk at 30 days for non-survivors and survivors among hospitalized SARS-CoV-2-positive veterans by indicated post-index medication use and race.

Post-Index		Non Survivors ^^^	Survivors ^^^	Multiplicity Corrected ^‡^	
Medication *		n (%)	n (%)	aRR (95% CI) ^†^	*p*-Value
Black (*N* = 3281)					
Vitamin D	Corticosteroids				
−	+	185 (45)	1089 (38)	1.0 Referent	---
−	−	215 (52)	1633 (57)	0.69 (0.55–0.86)	0.0009
+	+	5 (1)	70 (2)	0.44 (0.09–0.2.0)	0.29
+	−	5 (1)	79 (3)	0.34 (0.12–0.98)	0.047
White (*N* = 4291)					
Vitamin D	Corticosteroids				
−	+	329 (50)	1434 (40)	1.0 Referent	---
−	−	318 (48)	2020 (56)	0.65 (0.55–0.76)	<0.0001
+	+	9 (1)	76 (2)	0.50 (0.26–0.96)	0.036
+	−	6 (1)	99 (3)	0.29 (0.12–0.66)	0.0032

^^^ Non-referent group of the indicated comparison factor. * Systemic administration by mouth or intramuscular injection. ^†^ Adjusted for Age (≤60, 61–70, 71–80, >80), Alcohol Use Disorder (Yes, No), BMI (<18.5, 18.5–24.9, 25–29.9, ≥30), Charlson Comorbidity Index (0, 1–2, 3–4, 5^+^), Current Smoker (Yes, No), Location (Pacific-Mountain, Mid-West/Continental, East Coast), Sex (Male, Female), and Time (March, April–September). ^‡^ Subset analyses corrected for multiplicity using the Hochberg step-up procedure for multinomial comparisons. aRR = Adjusted relative risk; BMI = body mass index (kg/m^2^); CI = confidence interval.

**Table 4:** Adjusted risk at 30 days for non-survivors and survivors among non-hospitalized SARS-CoV-2-positive veterans by indicated post-index medication use and race.

Post-Index		Non Survivors ^^^	Survivors ^^^	Multiplicity Corrected ^‡^	
Medication *		n (%)	n (%)	aRR (95%CI) ^†^	*p*-Value
Black (*N* = 6534)					
Vitamin D	Corticosteroids				
−	+	16 (12)	811 (13)	1.0 Referent	---
−	−	116 (85)	5293 (83)	1.2 (0.68–2.0)	0.56
+	+	0 (0)	54 (<1)	∞	∞
+	−	5 (4)	239 (4)	0.93 (0.24–3.6)	0.92
White (*N* = 11,365)					
Vitamin D	Corticosteroids				
−	+	51 (15)	1233 (11)	1.0 Referent	---
−	−	284 (82)	9448 (86)	0.86 (0.42–1.7)	0.67
+	+	6 (2)	56 (1)	2.7 (0.77–9.2)	0.12
+	−	6 (2)	281 (3)	0.35 (0.10–1.2)	0.093

^^^ Non-referent group of the indicated comparison factor. * Systemic administration by mouth or intramuscular injection. ^†^ Adjusted for Age (≤60, 61–70, 71–80, >80), Alcohol Use Disorder (Yes, No), BMI (<18.5, 18.5–24.9, 25–29.9, ≥30), Charlson Comorbidity Index (0, 1–2, 3–4, 5^+^), Current Smoker (Yes, No), Location (Pacific-Mountain, Mid-West/Continental, East Coast), Sex (Male, Female), and Time (March, April–September). ^‡^ Subset analyses corrected for multiplicity using the Hochberg step-up procedure for multinomial comparisons. ∞ = non-convergent (zero cell). aRR = Adjusted relative risk; BMI = body mass index (kg/m^2^); CI = confidence interval.

**Table 5:** Adjusted risk for non-survivors and survivors among SARS-CoV-2-positive veterans by indicated factors and comparisons.

Stratum	Non-	Survivors ^^^	aRR	Non	Survivors ^^^	aRR	Effect (+|−)			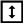 Stratum	
(Col/Row)	Survivors ^^^	n (%)	(95%CI) ^†^	Survivors ^^^	n (%)	(95%CI) ^†^	Δ_aRR_% ^♦^	*P*_Int_^†,¥^		*P*_ΔV_^†,^^⅄^	
	n (%)			n (%)				*MC’* ^‡^	*MC* ^‡^	*MC’* ^‡^	*MC* ^‡^
Post Index →	**Vitamin D (Yes versus No)**										
↓	Hospitalized +			Hospitalized −							
Corticosteroid +	15 (3)	149 (5)	0.51 (0.29–0.88)	6 (8)	113 (5)	2.5 (0.90–7.1)	−400	0.0071	0.028	0.057	0.12
Corticosteroid −	11 (2)	181 (5)	0.47 (0.26–0.84)	11 (3)	533 (3)	0.51 (0.25–1.03)	−8	0.88	0.88		
**Effect (+|−)**	**Δ_aRR_% ^♦^**		8			400					
	***P*_Int_^†,¥^**	***MC’* ^‡^**	0.86			0.012					
		*MC* ^‡^	1			0.036					
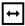 **Stratum**	***P*_ΔH_^†,⅄^**	***MC’* ^‡^**	0.062								
		*MC* ^‡^	0.062								
Post Index →	**Corticosteroid (Yes versus No)**										
↓	Hospitalized +			Hospitalized −							
Vitamin D +	15 (58)	149 (45)	3.0 (0.51–18)	6 (35)	113 (17)	6.2 (0.72–53)	−103	0.62	0.62	0.81	0.81
Vitamin D -	534 (49)	2615 (41)	1.5 (1.3–1.7)	69 (14)	2137 (12)	1.1 (0.81–1.4)	43	0.018	0.071		
**Effect (+|−)**	**Δ_aRR_% ^♦^**		98			479					
	***P*_Int_^†,¥^**	***MC’* ^‡^**	0.45			0.11					
		*MC* ^‡^	0.91			0.34					
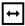 **Stratum**	***P*_ΔH_^†,⅄^**	***MC’***	0.53								
		*MC* ^‡^	1.0								

^^^ Non-referent group of the indicated comparison factor. ^†^ Adjusted for Age (≤60, 61–70, 71–80, >80), Alcohol Use Disorder (Yes, No), BMI (<18.5, 18.5–24.9, 25–29.9, ≥30), Charlson Comorbidity Index (0, 1–2, 3–4, 5^+^), Current Smoker (Yes, No), Location (Pacific-Mountain, Mid-West/Continental, East Coast), Race (White, Black, Other Race), Sex (Male, Female), and Time (March, April–September). ^♦^ Percentage change in aRR for hospitalized (+) versus non-hospitalized patients (−) on logarithmic scale. ^¥^ Unrestricted test for interaction on the additive scale. ^‡^ Uncorrected (***MC’***) | Corrected (*MC*) for multiplicity over 4 indicated strata within drug comparison using the Hochberg step-up procedure. ^⅄^ *p*-value for absolute difference (vertical/horizontal stratum effects, respectively). aRR = Adjusted relative risk; CI = confidence interval.

## Data Availability

These analyses were performed using raw data that are available only within the US Department of Veterans Affairs firewall in a secure research environment, the VA Informatics and Computing Infrastructure. To comply with VA privacy and data security policies and regulatory constraints, only aggregate summary statistics and results of our analyses are permitted to be removed from the data warehouse for publication. The authors have provided detailed results of the analyses in the paper. These restrictions are in place to maintain patient privacy and confidentiality.
